# CXorf61 is a target for T cell based immunotherapy of triple-negative breast cancer

**DOI:** 10.18632/oncotarget.4516

**Published:** 2015-07-29

**Authors:** Claudia Paret, Petra Simon, Kirsten Vormbrock, Christian Bender, Anne Kölsch, Andrea Breitkreuz, Özlem Yildiz, Tana Omokoko, Stefanie Hubich-Rau, Christoph Hartmann, Sabine Häcker, Meike Wagner, Diana Barea Roldan, Abderaouf Selmi, Özlem Türeci, Ugur Sahin

**Affiliations:** ^1^ TRON gGmbH, Translational Oncology at the University Medical Center, Johannes Gutenberg-University Mainz, Germany; ^2^ BioNTech Cell & Gene Therapies, An der Goldgrube 12, Mainz, Germany; ^3^ Experimental Oncology, Dpt. of Medicine III, Johannes Gutenberg-University, Mainz, Germany

**Keywords:** TNBC, CXorf61, CD8+ T cell epitope, T-cell receptor cloning, immunotherapy

## Abstract

Triple-negative breast cancer (TNBC) is a high medical need disease with limited treatment options. CD8+ T cell-mediated immunotherapy may represent an attractive approach to address TNBC. The objectives of this study were to assess the expression of CXorf61 in TNBCs and healthy tissues and to evaluate its capability to induce T cell responses.

We show by transcriptional profiling of a broad comprehensive set of normal human tissue that CXorf61 expression is strictly restricted to testis. 53% of TNBC patients express this antigen in at least 30% of their tumor cells. In CXorf61-negative breast cancer cell lines CXorf61 expression is activated by treatment with the hypomethylating agent 5-aza-2′-deoxycytidine.

By vaccination of HLA-A*02-transgenic mice with CXorf61 encoding RNA we obtained high frequencies of CXorf61-specific T cells. Cloning and characterization of T cell receptors (TCRs) from responding T cells resulted in the identification of the two HLA-A*0201-restricted T cell epitopes CXorf61_66–74_ and CXorf61_79–87_. Furthermore, by *in vitro* priming of human CD8+ T cells derived from a healthy donor recognizing CXorf61_66–74_ we were able to induce a strong antigen-specific immune response and clone a human TCR recognizing this epitope.

In summary, our data confirms this antigen as promising target for T cell based therapies.

## INTRODUCTION

Triple-negative breast cancer (TNBC) is any breast cancer that does not express the genes for estrogen receptor (ER), progesterone receptor (PR) and Her2/neu [1]. Triple negative is sometimes used as a surrogate term for basal-like breast cancer, which is a distinct, morphologically and molecularly defined entity. Due to lack of the receptors for targeted approaches, patients with TNBC have a poor outcome [2]. Conventional chemo- and radiation therapy have only limited efficacy in this cancer type underlining the urgent need for new treatment modalities.

There are indications that TNBCs are immunogenic cancers [3]. Therefore, T cell based treatment approaches, e.g. vaccines and adoptive T cell transfer are attractive for further exploration.

For such highly potent approaches tumor antigens with exquisite cancer cell-specificity are required. In this regard, a particularly interesting category of proteins of potential therapeutic utility are the so-called cancer/testis antigens (CTAs).

CTAs are a family of tumor antigens restricted to immune privileged sites such as testis and placenta but not expressed in any other organ [4]. For several CTAs spontaneous humoral as well as CD4+ and CD8+ T cell responses have been described in tumor patients [5–8]. Expression of CTAs in TNBC has been reported, with the MAGE-A family and NY-ESO-1 being the most prevalently expressed antigens [9]. Based on recent data the CTA CXorf61 (synonymous KK-LC-1, CT83) moved into the spot light as potential target in TNBC.

The CXorf61 sequence has first been identified by screening of a cDNA library derived from an allogeneic lung cancer cell line with an HLA-B*1507-restricted cytotoxic T lymphocyte (CTL) clone established from a lung cancer patient [10]. In a pilot screen of 20 normal tissues by RT-PCR (normal breast not included) testis tissue was the only one to express CXorf61 transcript [10]. Later, two independent approaches, one mining of massive parallel signature sequencing (MPSS) data [9], the other providing a survey of CTAs in the Cancer Genome Atlas (TCGA) RNA-seq datasets (http://cancergenome.nih.gov/) [11] revealed expression of *CXorf61* transcripts in the basal-like subtype of breast cancer. The *CXorf61* coding gene is located on chromosome Xq22 and consists of 113 amino acids. Its function and structure are largely unknown.

Until now, the notion that expression in normal tissues is restricted to testis is based on a narrow set of tissues, which were investigated by RT-PCR. Moreover, expression in breast cancers has been only shown on the transcript level and *in silico*. Protein expression has not been verified so far and it has not been positively excluded that CXorf61 is not in fact a breast tissue lineage marker.

One objective of this study was to verify the cancer/testis antigen pattern of CXorf61 expression and to assess CXorf61 expression in TNBC on the protein level. A second objective was to prototypically assess the capability of CXorf61 to induce T cell responses *in vitro* and *in vivo*.

Our findings strongly support the utility of CXorf61 for antigen-specific T cell based approaches and warrant further exploration.

## RESULTS

### Frequent expression of CXorf61 mRNA in TNBC samples and absence from the vast majority of normal human tissue types

**Figure 1:**
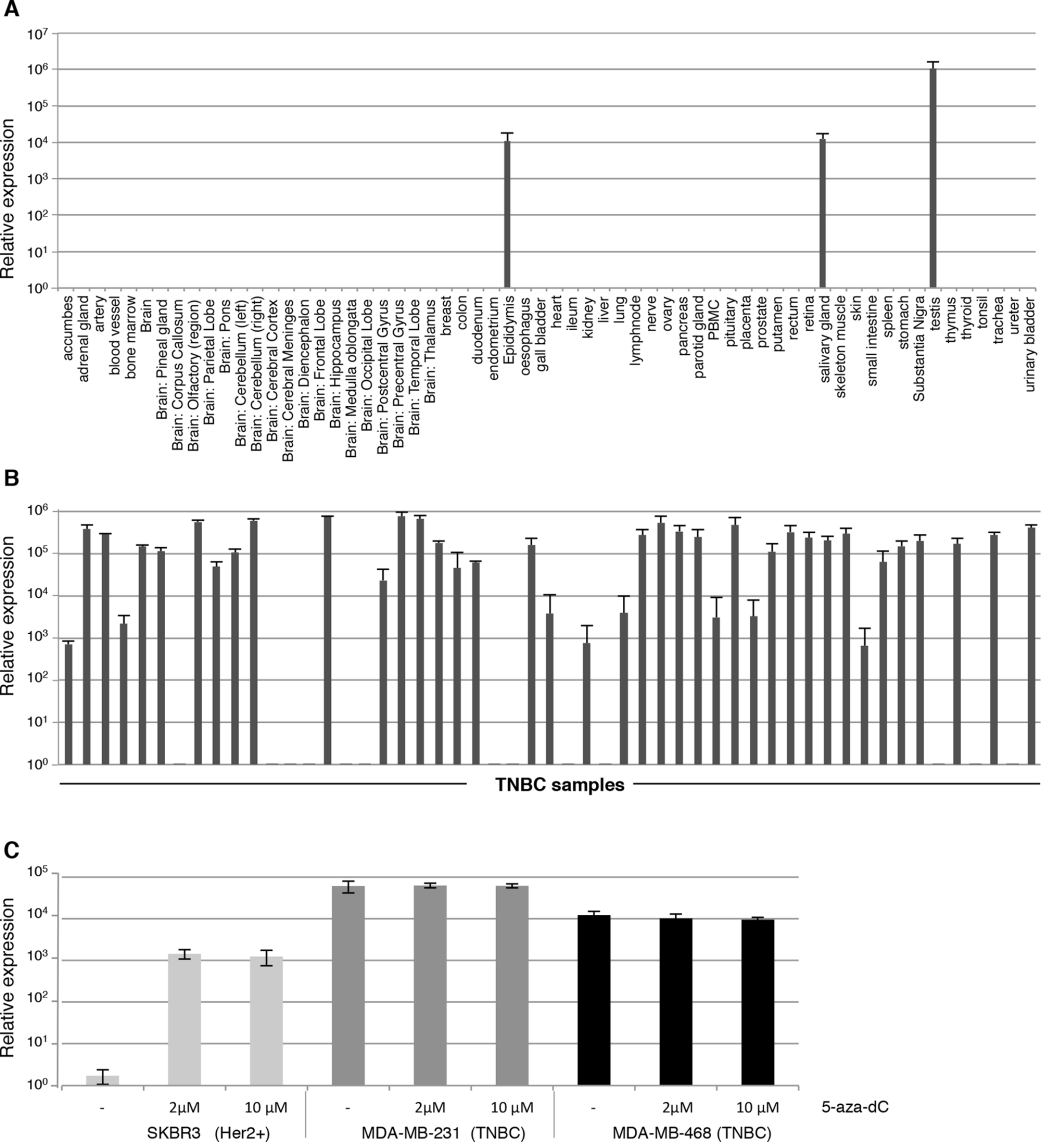
Frequent expression of *CXorf61* mRNA in TNBC samples and absence from the vast majority of normal human tissue types *CXorf61* expression was analysed by qRT-PCR using the BioMark™ HD system on 62 normal tissue types **A.** and 53 TNBC samples **B, C.** Expression of *CXorf61* in human breast cancer cell lines by qRT-PCR without (−) or after addition of 5-aza-dC. After normalization to the housekeeping gene *HPRT1*, the relative quantification value was expressed as 2^−ΔΔCt^. Expression analysis was done in triplicates. Standard deviation is indicated.

To verify the restricted expression of CXorf61 in normal tissues, we used the Fluidigm technology, which allows highly reproducible measuring of mRNA levels by qRT-PCR in up to 96 samples in parallel [12]. *CXorf61* expression was analyzed in a broad and diversified panel of 62 normal tissue types. Robust expression was found in testis only (rel. expr. 10^6^). Weak signals two magnitudes lower in intensity were measured in salivary gland and epididymis (rel. expr. 10^4^) (Fig. 1A). In all other tissue including normal breast, thymus and highly toxicity-relevant organs such as heart muscle, lung, liver, and a variety of brain areas expression was below detection level.

Next we analyzed *CXorf61* mRNA expression in TNBC samples. The vast majority of samples were of ductal histology, poorly differentiated, of T2 size and were derived from localized disease (Table 1A), representing the typical TNBC population at the time of diagnosis [13, 14]. Expression of the *CXorf61* transcript was detected in 40 of 53 (75%) of the TNBC samples (Fig. 1B, Table 1B and Supplementary Table S1). Half of the analyzed TNBC samples had relative expression levels above 10^5^.

**Table 1A:** Clinicopathological characteristics of breast cancer patients in the tested cohort (*n* = 63)

		*n*	%
Age at diagnosis	Median (SD)	53 (range 31–85)	
Grade	1	1	2
	2	18	29
	3	42	67
	Unknown	2	3
Histology	Ductal	50	79
	Lobular	2	3
	Unknown	11	17
Size	T1	3	5
	T2	49	78
	T3	8	13
	T4	2	3
	Unknown	1	2
Lymphnode status	pN0	41	65
	pN1/2/3	21	33
	Unknown	1	2

**Table 1B:** Frequency of CXorf61 expression

	Number of positive samples/analysed samples	%
qRT-PCR positive	40/53	75
IHC positive, any	11/17	65
IHC positive, >30%	9/17	53
IHC positive, >50%	7/17	42

Down-regulation of CTAs due to promoter methylation may account for heterogeneous expression in tumor tissues. It has previously been shown that the CXorf61 promoter is highly hypomethylated in basal tumors [11]. We analyzed the effect of promoter methylation on *CXorf61* expression by treating TNBC cell lines MDA-MB-231 and MDA-MB-468 [15], and the HER2-positive cell line SKBR3 [16] with the hypomethylating agent 5-aza-dC. We found that *CXorf61* is highly expressed in the two triple negative cell lines but below detection level in the HER2 positive cell line SKBR3 (Fig. 1C). By culturing SKBR3 in 5-aza-dC supplemented medium, however, *CXorf61* transcript was switched on and detectable at a relative expression level of 10^3^ fold. In the two cell lines with constitutively high expression of *CXorf61* hypomethlyation did not appear to have an effect on *CXorf61* expression levels.

In summary our findings confirm and further extend transcriptional data supporting that *CXorf61* is a cancer testis antigen. *CXorf61* transcripts are highly and frequently expressed in TNBC tissues but are absent from any other normal tissue except for testis. Hypermethylation of *CXorf61* promoter may be the primary inactivating event in tumour cells not expressing the transcript.

### Robust protein expression levels of CXorf61 in primary TNBC, TNBC cell lines and normal testis

**Figure 2:**
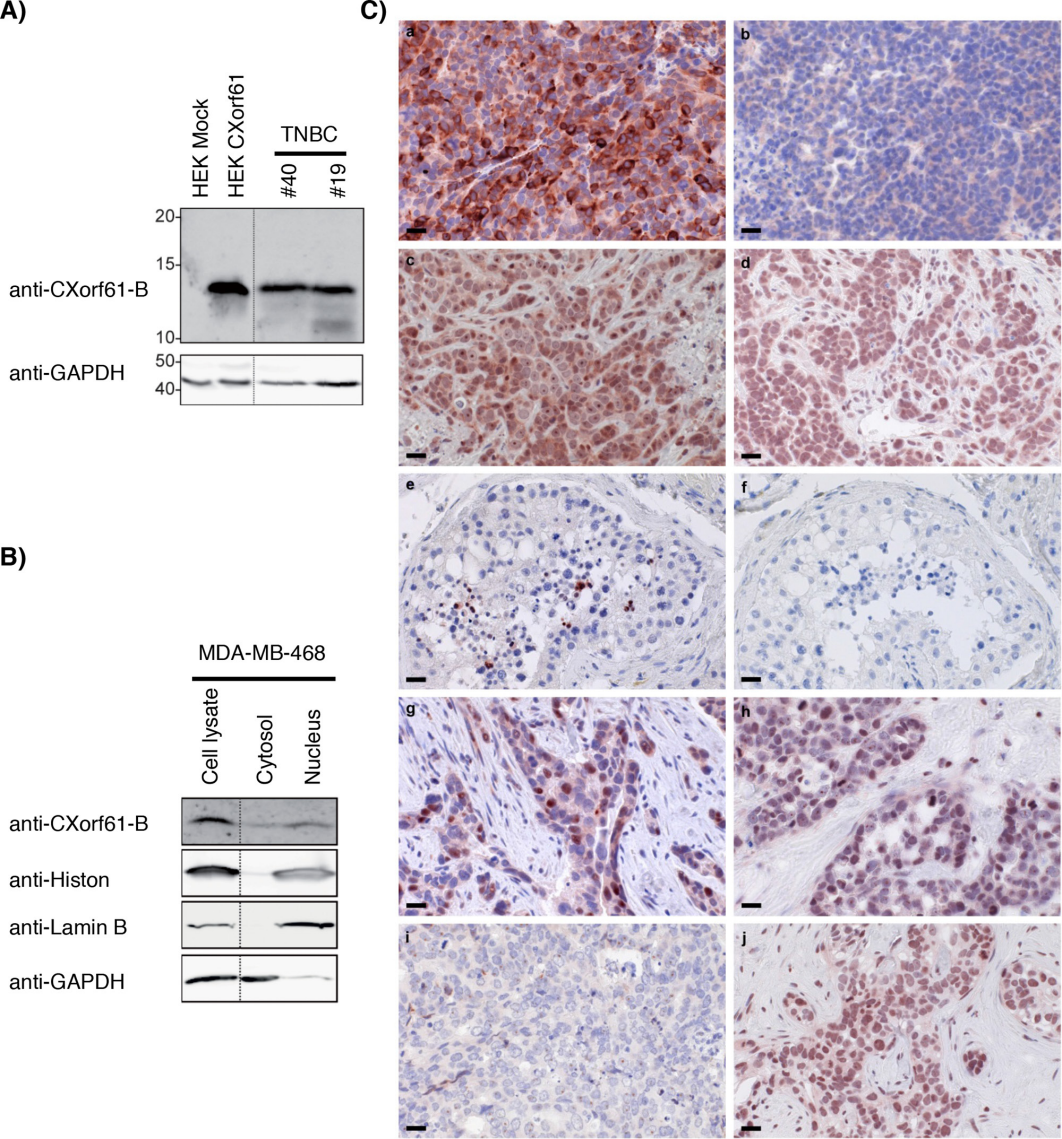
Robust expression of CXorf61 protein in primary TNBC, TNBC cell lines and normal testis **A.** CXorf61 protein expression was analyzed with antibody anti-CXorf61-B in the lysates of 2 TNBC samples (patients # 40 and 19, Supplementary Table S1). GAPDH was used as loading control. Positive control: lysate of HEK 293T transfected with a plasmid coding for CXorf61. Negative control: HEK 293T transfected with empty vector. **B.** Nuclei and cytosol isolated from the MDA-MB-468 cell line were analysed by Western Blot with the CXorf61 specific antibody anti-CXorf61-B or antibodies against different cellular compartments (Histon, Lamin B, GAPDH). **C.** Staining of tissue sections by immunohistochemistry with antibody anti-CXorf61-A. Tissues were obtained by xenografting HEK 293T-CXorf61 (a) or HEK 293T-mock (b), MDA-MB-468 cells (c) and MDA-MB-231 cells (d) in mice. Human testis (e) and 4 TNBC tissues (g-j) were stained. Negative control: staining of the testis without any primary antibody (f). Tissues g-j correspond to patients # 40, 56, 48 and 62 (Supplementary table S1). Scale bar = 20 μM.

To assess whether the high transcript levels of CXorf61 in TNBC translate into robust expression of the protein, Western blot analysis with polyclonal serum anti-CXorf61-B was performed. A strong signal, compatible with the predicted size of 13 KDa, was detected in lysates of two primary TNBC specimens as well as in CXorf61-transfected HEK cells (HEK CXorf61), but not in mock transfected HEK cells (HEK Mock) (Fig. 2A). Analysis of subcellular fractions of the TNBC cell line MDA-MB-468 with the same detection system revealed presence of the CXorf61 protein in the nucleus as well as in the cytoplasmic fraction (Fig. 2B).

As polyclonal serum anti-CXorf61-B does not function in immunohistochemistry on FFPE tissue, antibody anti-CXorf61-A was generated in rabbit and affinity purified. Specificity of this antibody was confirmed by Western Blot analysis and detection of the 13kDA band in HEK-CXorf61 but not HEK mock cells (Supp. Fig. 1). In immunohistochemistry, mouse xenograft tumor tissue sections derived from constitutively CXorf61-expressing MDA-MB-468 and MDA-MB-431 cells and HEK-CXorf61 were robustly stained, whereas HEK-mock derived xenografts were not stained (Fig. 2C), further supporting the specificity of the antibody.

In testis tissue sections, the only normal tissue in which transcript had been detected, strong expression of CXorf61 was found on fully mature spermatids only (Fig. 2Ce). In tissue sections of TNBC, CXorf61 positive cells were found in 11 of 17 of TNBC specimen tested. Individual tumour samples were heterogeneous in terms of fraction of positive cells. 9 of 17 patients (53%) expressed CXorf61in at least 30% of the tumour cells. In 7 of 17 patients (42%) at least 50% of the tumour cells were stained (Fig. 2C, Table 1B, and Supplementary Table S1). In all cases staining was of strong intensity and predominantly localized to the nucleus and cytoplasm.

In summary, our data indicates that robust levels of CXorf61 protein are expressed in tumours of a significant proportion of TNBC patients.

### Cloning of TCRs from CXorf61-specific T cells induced by immunization of HLA-transgenic mice and identification of HLA-A*02-restricted epitopes

**Figure 3:**
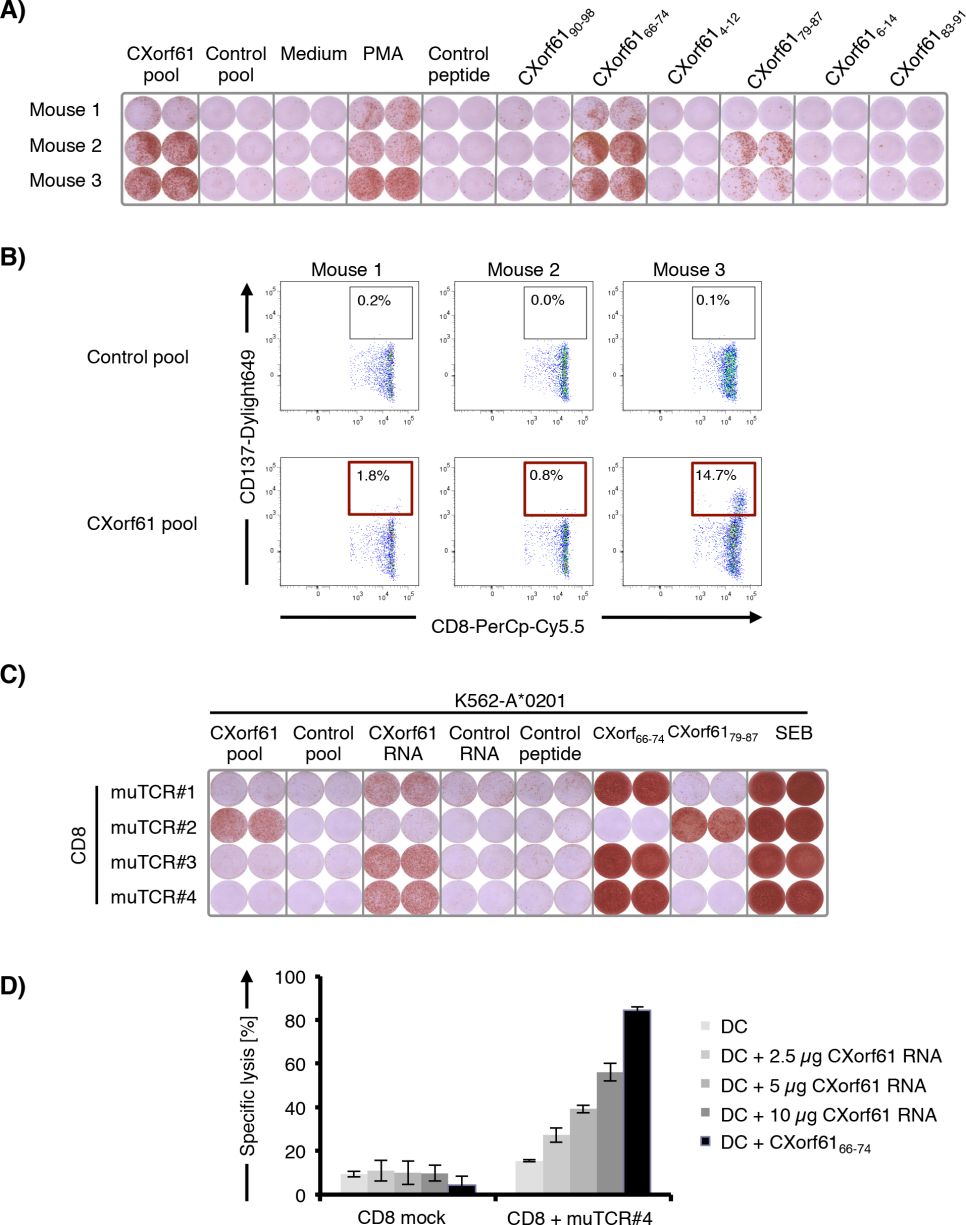
Cloning of murine TCRs from CXorf61-specific T cells induced by immunization of HLA-transgenic mice and identification of HLA-A*02-restricted epitopes **A.** Detection of CXorf61-specific T cells after 5 rounds of immunization of HLA-A*0201-transgenic mice with CXorf61 encoding IVT RNA. Spleen cells from three mice were analyzed for reactivity against CXorf61 overlapping peptide pool (CXorf61 pool) or predicted HLA-A*0201-binding CXorf61-derived peptides by IFNy-ELISPOT assay. **B.** FACS sorting of CXorf61-specific murine CD8+ T cells from spleen cells of immunized HLA-A*0201-transgenic mice after *in vitro* restimulation with overlapping peptide pools. Cells were gated on CD3+/CD8+ lymphocytes. Single CD8+/CD137+ T cells were isolated **C.** Specificity testing of murine TCRs isolated from CD8+ T cells of CXorf61-immunized mice. CD8+ T cells of a HLA-A*0201-positive healthy donor were transfected with TCR-α/β chain RNAs and tested for recognition of K562-A*0201 transfected with CXorf61 IVT RNA or pulsed with CXorf61 peptide pool or HLA-A*02 binding peptides by IFNγ-ELISPOT. **D.** OKT3-activated CD8+ T cells from a healthy donor were transfected with the murine TCR#4 IVT RNA and co-cultured with autologous immature DCs transfected with CXorf61 IVT RNA or loaded with CXorf61_66–74_. Specific killing was analyzed by luciferase cytotoxicity assay. Positive controls: Phorbol-12-myristate-13-acetate treated spleen cells (PMA); Staphylococcus Enterotoxin B treated cells (SEB); negative controls: spleen cells without stimulus (medium); irrelevant HLA-A*0201-restricted peptide PLAC1_31–39_ (control peptide); pool of overlapping 15mer peptides representing the irrelevant antigen HIV-gag (control pool); IVT RNA encoding the irrelevant antigen TPTE (control RNA), T cells transfected without TCR RNA (CD8 mock).

To analyze the *in vivo* immunogenicity of CXorf61 we immunized HLA-A*02 transgenic mice 5 times with IVT RNA encoding CXorf61. Induction of CXorf61-specific CD8+ T cells was assessed by an *ex vivo* IFNγ-ELISPOT assay. As antigens in the ELISPOT assay we used either a pool of overlapping 15-mer peptides spanning the entire CXorf61 sequence or six synthetic 9-mer peptides predicted to bind to HLA-A*0201 by the SYFPEITHY algorithm (Supplementary Table S2) [17]. Spleen cells of immunized mice were analyzed for reactivity against each of these CXorf61 derived antigens and appropriate control antigens (Fig. 3A). Significant frequencies of CXorf61-specific T cells as measured with the peptide pool were particularly strong in two of the mice. T cell reactivity was found to be directed against peptide CXorf61_66–74_ and CXorf61_79–87_.

To characterize the T cell response induced against CXorf61, we restimulated splenocytes from immunized mice *in vitro* with the CXorf61 overlapping peptide pool. CXorf61-specific CD8+ T cells with activation-induced upregulation of CD137 were isolated by flow cytometry (Fig. 3B) and TCRs were cloned from single cells and sequenced. IVT RNA encoding full-length CXorf61-TCR α/β chains of these TCRs was generated. For functional validation of the cloned TCRs, CD8+ T cells of a HLA-A*0201-positive healthy donor were transfected with TCR-α/β chain RNAs and tested in IFNγ-ELISPOT. As target cells K562-A*0201 cells transfected with CXorf61 IVT RNA or pulsed with CXorf61 overlapping 15mer peptides or with the HLA-A*0201 binding peptides CXorf61_66–74_ and CXorf61_79–87_ were used (Fig. 3C). One of the TCRs recognized epitope CXorf61_79–87_, however only on peptide-pulsed and not transfected target cells. The three remaining murine TCRs recognized epitope CXorf61_66–74_ pulsed on target cells as well as processed from transfected CXorf61 IVT RNA, indicating that this epitope can be generated from constitutively expressed CXorf61.

Next we wanted to analyze if CXorf61_66–74_ is efficiently processed and presented by human dendritic cells, which are pivotal for priming and sustaining T cell responses. Pre-activated CD8+ T cells of a healthy donor were transfected with IVT RNA encoding muTCR#4, and analyzed for lysis of autologous DCs transfected with increasing amounts of CXorf61 IVT RNA using a luciferase-based cytotoxicity assay. TCR-engineered T cells efficiently and specifically lysed CXorf61-expressing DCs in a dose-dependent, saturable manner. No lysis was mediated by CD8+ T cells not engineered with TCR RNA or of CXorf61 negative DCs (Fig. 3D).

Taken together, we describe two novel CXorf61 epitopes presented by a frequent HLA allelotype and capable of inducing strong immune responses, for one of which we show that it can be processed and presented. Moreover, we demonstrate that TCRs cloned from these CXorf61-specific T cells are capable of recognizing and lysing antigen-expressing targets very efficiently.

### Induction of human CXorf61-specific T cells *in vitro* and discovery of a CXorf61-specific human TCR

**Figure 4:**
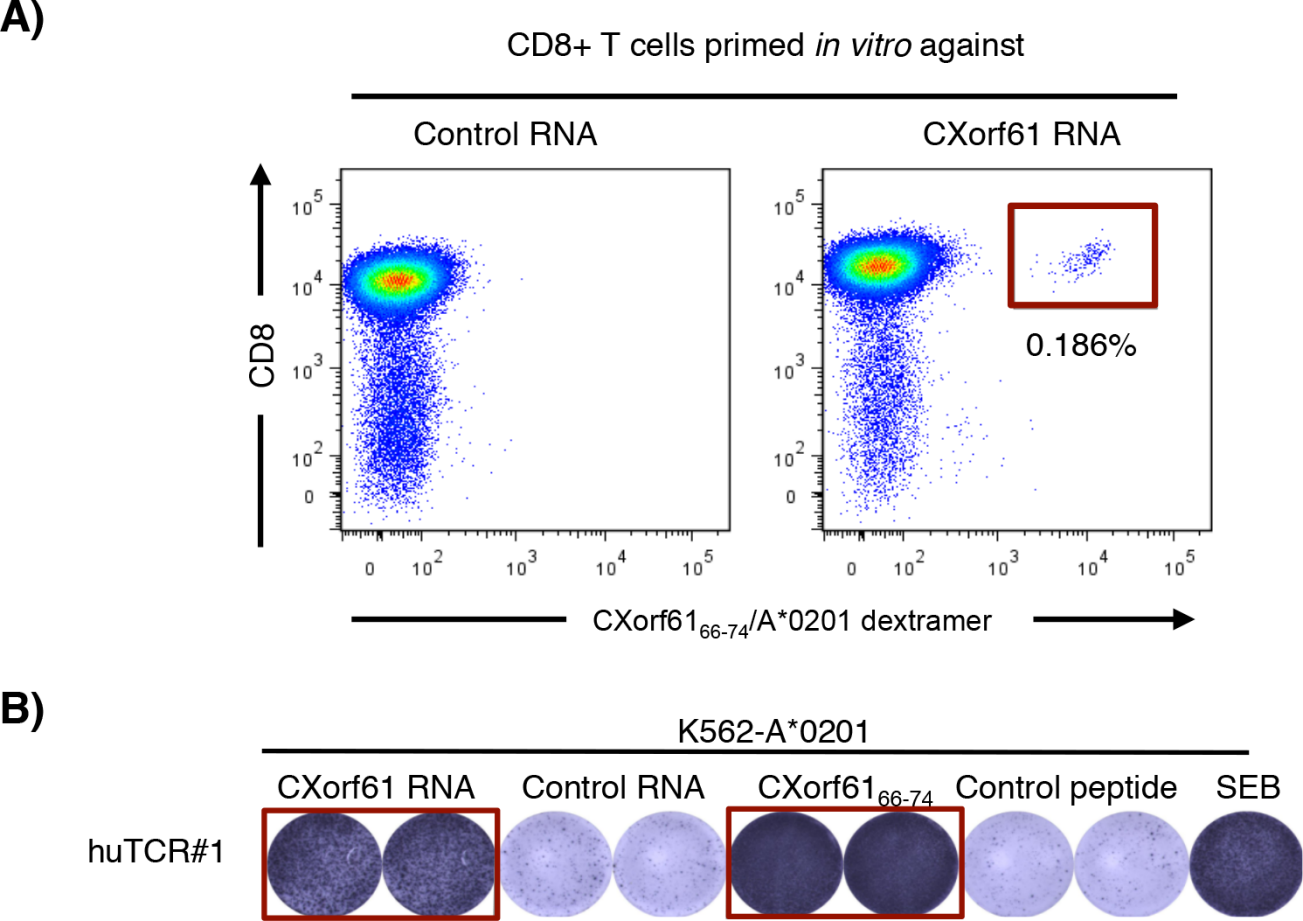
Induction of human CXorf61-specific T cells *in vitro* and cloning of a CXorf61-specific human TCR **A.** CD8+ T cells of a healthy HLA-A*0201 expressing donor were primed using autologous mDC transfected with CXorf61 RNA. After three rounds of stimulation CXorf61-specific T cells were detected by CXorf61_66–74_/A*0201 dextramer staining and isolated by flow cytometry for TCR cloning. **B.** Specificity testing of a TCR isolated from human CD8+ T cells sensitized against CXorf61. CD8+ T cells transfected with the cloned TCR chains were tested by IFNγ-ELISPOT for recognition of K562-A*0201 cells either transfected with CXorf61 IVT RNA or loaded with CXorf61_66–74_; negative controls: irrelevant RNA CLDN6 (control RNA); irrelevant peptide PLAC1_31–39_ (control peptide); positive control: SEB treated cells.

To analyze the immunogenicity of CXorf61 in the context of human T cells, we primed CD8+ T cells of a healthy HLA-A*0201 expressing donor *in vitro* using autologous DCs transfected with CXorf61 or control IVT RNA. After 3 rounds of stimulation, CXorf61-specific T cells were detected using the CXorf61_66–74_/A*0201 dextramer (Fig. 4A). A defined population of 0.186% of lymphocytes within the sample primed with CXorf61-transfected DCs was stained by a CD8-specific antibody and the CXorf61_66–74_/A*0201 dextramer. No CXorf61-specific T cells were detected in the control sample where T cells were primed against an irrelevant antigen.

To verify the specificity of the *in vitro* primed CXorf61-specific T cells we cloned the corresponding human TCR genes from single cells. CD8+ T cells of the healthy donor were transfected with TCR-α/β chain encoding IVT RNAs and tested in IFNγ-ELISPOT for recognition of K562-A*0201 target cells pulsed with the CXorf61_66–74_ epitope or transfected with CXorf61 RNA. Thereby, we identified a human TCR specifically recognizing CXorf61_66–74_ peptide pulsed as well as IVT RNA transfected DCs (Fig. 4B).

These data confirm that CD8+ T cells directed against CXorf61_66–74_ can be induced *in vitro* indicating that CXorf61-specific T cells are not deleted during T cell maturation as a consequence of central tolerance mechanisms.

## DISCUSSION

This study was conducted to assess utility of CXorf61 for T cell based immunotherapy in triple-negative breast cancer, a disease of high medical need.

Key findings relate to the expression of CXorf61 in normal and tumor tissues. Expression of CTAs in normal tissues is not as restricted as generally thought. A recent survey classified 153 CTAs in three groups: truly testis-restricted, testis/brain-restricted and in a testis-selective group that show additional expression in somatic tissues [18]. For e.g. MAGE family members low but clinically relevant expression in the brain was originally overlooked [19]. Previous studies have categorized CXorf61 as CTA based on assessing CXorf61 transcript expression in a limited number of human normal tissue types [9–11]. We show by investigating a much broader and comprehensive tissue set that *CXorf61* is indeed restricted to the testis with very low expression in epididymis and salivary gland, both non-life essential organs. Moreover, our study tests and excludes expression in a number of tissues which have not been investigated for CXorf61 so far, e.g. normal breast and a diversity of brain areas.

Published data on CXorf61 in human cancers was so far based on *in silico* data and transcript profiling without data on protein expression. We show for the first time that CXorf61 is expressed as a 13kDA protein in primary human cancers and cancer cell lines predominantly localized in the nucleus and cytoplasm. We generated an antibody for staining of FFPE tissue and show that there is some heterogeneity in TNBC tumor tissues. However, more than half of the patients express CXorf61 robustly on the protein level in more than a third of their tumor cells. Thus, the frequency of CXorf61 expression in TNBC is even higher than that of NY-ESO-1 (16–18%) and MAGE-A3 (26%), two CTAs pursued for immunotherapy of TNBC [20, 21]. Given the fact that for most T cell based treatment approaches one would expect bystander activity by reshaping of the tumor microenvironment, we believe that also patients with heterogeneous expression would be eligible for CXorf61 therapies. The more, as we show that by using hypomethylating agents, CXorf61 expression can be switched on in non-expressing tumor cells. Several clinical trials employ a hypomethylation strategy to re-express silenced targets in breast carcinomas [22].

In even a higher percentage of TNBCs (75%), CXorf61 is detected by RT-PCR, most likely because protein levels are below detection level of the used assays. Further investigations are warranted to answer which amount of protein is needed for recognition of processed epitopes on MHC complexes by T cells.

Other key findings of our study refer to the capability of CXorf61 to induce antigen-specific T cell responses. CXorf61 was identified in 2006 as a target recognized by a HLA-B*1507-restricted cytotoxic T lymphocyte (CTL) clone and the respective peptide epitope was determined. We discovered two epitopes capable of binding to HLA-A*0201, a frequent HLA allelotype, and of inducing profound stimulation and expansion of cytokine secreting CD8+ T cells in mice after vaccination. For CXorf61_66–74_, we show processing from endogenously expressed CXorf61. T cell responses induced against this peptide kill efficiently. Moreover, we present two molecularly defined TCRs against this epitope. One is derived in HLA-A*0201-transgenic mice immunized with CXorf61-encoding IVT RNA. The other arose by pre-sensitization of human PBMCs *in vitro*, indicating that immune responses directed against CXorf61 are not impaired by central tolerance.

These data exemplifies that using the technology platform for TCR cloning from single cells, we described elsewhere in more detail [7], it should be feasible, to further extend the repertoire of vaccine candidate peptide epitopes and TCR reagents for this CTA.

The identified epitopes are not found in other proteins (data not shown) excluding cross reactivity with other antigens that may be expressed in vital organs, as recently described for MAGE-A3 [19]. Noteworthy, the only previously described CXorf61 B*1501-restricted T cell epitope [10] is at aa position 76–84, thus clustering with the epitopes, we describe here, suggesting the importance of the region between aa 66–87 for the immunogenicity of CXorf61.

In conclusion, our study demonstrates that CXorf61 fulfils the requirements of an ideal target for cancer immunotherapy as it is cancer–cell selective, immunogenic and expressed at high level and frequency in TNBC tumors. Hence, further exploration of CXorf61 is warranted.

## MATERIALs AND METHODS

### Tissue samples, cell lines, PBMCs, monocytes and dendritic cells (DCs)

Tissues and/or RNAs of normal and TNBC tissues were sourced from commercial vendors. 46 TNBC tissues were available as frozen, 10 as Formalin-fixed paraffin-embedded (FFPE) tissues and 7 as both. Cell lines were obtained from ATCC and cultured in Dulbecco's modified Eagle's medium (DMEM), 10% fetal calf serum. K562 cells stably transfected with HLA-A*0201 (K562-A*0201) were cultured under standard conditions in the presence of 1 mg/ml geneticin. CXorf61-transfected HEK 293T was generated by standard methods using a cDNA containing the full length open reading frame of human CXorf61 subcloned into pCR3.1 (Invitrogen, Darmstadt, Germany). For some experiments cell were cultured in 6-well plates and treated daily with medium supplemented with 2 or 10 μM 5-aza-2′-deoxycytidine (5-aza-dC, Sigma Aldrich) and harvested after 72 h of treatment.

For some experiments, tissue was generated from human cancer cell lines by xenografting into immunocompromised mice and grown for up to 10 days until nodules were well palpable. Tumors were fixed with 4% buffered formaldehyde-solution (Roti-Histofix, Carl Roth) and embedded into paraffin (Paraplast, Carl Roth).

PBMCs were isolated by Ficoll-Hypaque (Amersham Biosciences) density gradient centrifugation from buffy coats. CD8+ T cells were purified from PBMC by positive magnetic cell sorting (Miltenyi Biotec). Monocytes were enriched with anti-CD14 microbeads (Miltenyi Biotec). DCs were obtained as described previously [23]. For some experiments primary cells were electroporated with *in vitro* transcribed (IVT) RNA as previously described [23].

### qRT-PCR

RNA isolation was conducted using the RNeasy Lipid Tissue Mini Kit procedure for frozen tissues and the RNeasy Mini Kit for cell lines (Qiagen). RNA was converted to cDNA by using PrimeScript RT Reagent Kit with gDNA Eraser (Takara Bio Inc.). qRT-PCR was performed using the ABI PRISM 7300 detection system and software (Applied Biosystems with QuantiTect SYBR green Kit (Qiagen)) or the BioMark™ HD system (Fluidigm). For qRT-PCR analysis using the BioMark™ the samples and assays were prepared and analyzed according to the “Fast Gene Expression Analysis Using EvaGreen^®^ on the BioMark™ or BioMark HD SystemFluidigm^®^ Advanced Development Protocol 37”. 96.96 Gene Expression Dynamic Array IFCs were loaded using the IFC Controller HX. Primers 5′-GTGTGCCTTGAT TGTCTTCTGG and 5′-CCTGGCTATTGAGTGTGGG were used for the detection of for *CXorf61* and 5′- TGACACTGGCAAAACAATGCA and 5′-GGTCCTTTTCACCAGCAAGCT for the detection of *Hypoxanthin-Phosphoribosyl-Transferase 1 (HPRT1)*. For analysis with the BioMark™ HD system, the cut-off for reliable Ct values was set to 21 and higher values were replaced with 30; only tissues with a Ct value for *HPRT1* less than 15 were accepted for the analysis. After normalization to the housekeeping gene *HPRT1*, the relative quantification value was expressed as 2^−ΔΔCt^. The calibrator was calculated as the maximal number of cycles used in the PCR (30 for the BioMark and 40 for the ABI PRISM system) minus the mean of the HPRT1 Ct values, resulting in a value of 18, 2 for the BioMark system and 16 for the ABI PRISM system

### *In vivo* priming of CXorf61-specific T cells in HLA A2.1/DR1 mice

T cells of HLA A2.1/DR1 mice [24] were primed *in vivo* against CXorf61 by five intranodal immunizations with CXorf61-encoding IVT RNA [25]. For intranodal immunizations, mice were anesthetized, the inguinal lymph node was surgically exposed, 10 μL RNA (20 μg) diluted in Ringer's solution and Rnase-free water were injected slowly using a single-use 0.3-ml syringe with an ultrafine needle (31G, BD Biosciences).

### *In vitro* priming of human CD8+ T cells

2×10^6^ CD8+ T cells were cocultured together with 2×10^5^ IVT RNA transfected mDC in 24-well plates (Costar). mDCs were transfected with a mixture of adjuvant RNAs encoding CD40 ligand, CD70 and constitutively active TLR4 [26] as well as with IVT RNA encoding either CXorf61 or an irrelevant control antigen. Cells were cultured in RPMI 1640 Glutamax, 100 U/ml penicillin, 100 μg/ml streptomycin, 1 mM sodium pyruvate, nonessential amino acids, and 5% heat-inactivated human AB serum (all from Invitrogen). IL-7 (5 ng/ml; Miltenyi) was added on day 0 and 50 U/ml IL-2 (Proleukin, Novartis) after 2 days and then on every third subsequent day. The second and third stimulation of primed T cells was made after 7 and 14 days using thawed IVT RNA-transfected mDCs. After 3 rounds of stimulation cells were analyzed by CXorf61_66–74_/HLA*02 dextramer staining (Immudex).

### Flow cytometry based isolation of single CXorf61-specific CD8+ T cells

Spleens of immunized HLA A2.1/DR1 mice were mechanically disrupted and the cell suspensions were obtained with a cell strainer (40 μm). The splenocytes were washed with PBS centrifuged and resuspended in a hypotonic buffer for lysis of the erythrocytes. For antigen-specific restimulation 2.5×10^6^/well spleen cells were seeded in a 24-well plate and pulsed with a pool of overlapping peptides encoding *CXorf61* or a control antigen (HIV-gag). After 24 h incubation cells were harvested, stained with a FITC-conjugated anti-CD3 antibody, a PerCP-Cy5.5-conjugated anti-CD8 antibody and a Dylight-649-conjugated anti-CD137 antibody (all antibodies were purchased from eBioscience).

*In vitro* induced human CXorf61-specific CD8+ T cells were isolated after staining with an Allophycocyanin-labeled CD8-specific antibody (BD Biosciences) and a Phycoerythrin-labeled CXorf61_66–74_/HLA*02 dextramer (Immudex).

Sorting was conducted on a BD FACS Aria flow cytometer (BD Biosciences). One T cell per well was harvested in a 96-well V-bottom-plate (Greiner Bio-One) containing either human CCD-1079Sk in the case of murine T cells or NIH-3T3 cells in the case of human T cells as feeder cells, centrifuged at 4°C and stored immediately at −80°C.

### Cloning of human and murine TCRs

Cloning of TCR genes from single human T cells was performed as previously described [7]. In brief, total RNA extracted from sorted cells via the Micro RNeasy Kit (Qiagen) was used for template-switch cDNA synthesis with Mint Reverse Transcriptase (Evrogen) followed by preamplification using Pfu Ultra Hotstart DNA Polymerase (Agilent). Aliquots of the resulting cDNAs were used for Vα-/Vβ gene-specific multiplex PCRs. Products were analyzed using Qiagen's capillary electrophoresis system. Samples with bands at 400–450 bp were size fractioned on agarose gels and the bands excised and purified using a Gel Extraction Kit (Qiagen). Purified fragments were sequenced and the respective V-D-J junctions analyzed using the IMGT/V-Quest tool [27] DNAs of novel and productively rearranged TCR chains were NotI-digested and cloned into IVT vectors containing the appropriate backbones for complete TCR-α/β chains [7].

Cloning of TCR genes from single murine T cells was performed analogously using a panel of murine Vα- and Vβ-specific forward oligos and a murine Cα- as well as a murine Cβ-specific reverse oligo for amplification. The forward oligos were designed as described in [7] by aligning all functional murine V genes as listed in the IMGT database [28]. IVT vectors containing the gene-optimized murine Cα, Cβ1 and Cβ2 genes, respectively, all obtained from Eurofins Genomics, were constructed using the previously described pST1-sec-insert-2βgUTR-A(120)-Sap1 backbone [23]. Specific V(D)J PCR products were introduced into these cassettes via NotI and EcoRV sites to yield plasmids encoding full length murine TCR chains (referred to as pST1-mTCRα/β-2βgUTR-A(120)).

### Cellular assays

The ELISPOT used to analyze antigen-specific IFNγ secretion of TCR-engineered T cells was performed as described previously [7]. For measuring cytotoxicity, 1×10^4^ DCs cotransfected with luciferase-encoding IVT RNA and titrated amounts of CXorf61 encoding RNA were co-cultured with TCR-transfected T cells for 3 h. D-Luciferin (BD Biosciences) was added to the cells. One hour later luminescence was measured using a Tecan Infinite M200 reader (Tecan). Cell killing was calculated by measuring the reduction of total luciferase activity. Viable cells were measured by the luciferase-mediated oxidation of luciferin. Specific killing was calculated by following equation:

$$\text{Specific killing in }\%\;=\;100-\left[\frac{(\text{sample}-\text{complete lysis}}{(\text{max viable cells}-\text{complete lysis}}\times 100\right]$$

### Generation of a polyclonal CXorf61 antibody (anti-CXorf61-A)

Rabbits were immunized with a CXorf61-peptide corresponding to amino acids 67–80 at Charles River Laboratories (Sulzfeld, Germany). Crude rabbit sera were further purified by affinity chromatography on immunisizing peptides using the Äcta Purifier 900 system (GE Healthcare, Munich, Germany) and an acidic gradient.

### Preparation of subcellular compartments

Nuclear and cytoplasmic extracts were generated by swelling 1 × 10^7^ cells for 30 min in 500 μl hypotonic buffer N (10 mM HEPES (pH 7.5), 2 mM MgCl_2_, 25 mM KCl) followed by douncing. Afterwards 250 mM Sucrose was added and nuclei were pelleted by centrifugation at 700 x g for 10 min at 4°C. Cytoplasma containing supernatants were collected and nuclei were washed once in hypotonic buffer N containing 250 mM Sucrose.

### Immunohistochemistry

3 μm sections of FFPE tissues were generated using a rotary microtome (Leica, RM2255), deparaffinized and re-hydrated in the bi-linear stainer Stainmate Max (Thermofisher) followed by heat-induced epitope retrieval in 10mM citric buffer pH6 with 0, 05% Tween20 (10min at 120°C). Endogenous peroxidases were quenched using 0, 3% H_2_O_2_ solution in PBS (Carl Roth) and blocked with 10% goat serum in PBS (PAA). The CXorf61 antigen was detected by incubation with a purified polyclonal antibody (anti-CXorf61-A) at 4°C followed by the incubation with the BrightVision polymer HRP-conjugated anti-rabbit secondary antibody (Immunologic). Binding reactions were visualised using the Vector NovaRED kit (Vector Laboratories) followed by haematoxylin counterstaining (Carl Roth), dehydration and mounting. Analysis and documentation were performed using either the AxioImagerM2 (Zeiss) or the Axio Scan (Zeiss).

### SDS-PAGE and Immunoblotting

Fresh frozen tissues were lysed using the TissueLyser (Qiagen,) and 4x SDS-lysis buffer (250mM Tris/HCl, 34% (w/v) Glycerin, 8.2% (w/v) SDS, 5% (v/v) Δ-Mercaptoethanol, pH 6.8) supplemented with protease inhibitor cocktail (Roche). Total cell extracts were generated by scraping cells from the cell culture plate applying a 4x SDS-lysis buffer. After sonication, protein concentration of samples was measured by OD280. Equal amounts were loaded onto SDS-PAGE gels [29] and electrotransferred onto nitrocellulose membrane (0.1 μm, GE Healthcare). Immunostaining was performed with primary antibody followed by detection with horseradish-peroxidase conjugated antibodies (Pierce). A Polyclonal antibody against CXorf61 (anti-CXorf61-B) was purchased from Sigma-Aldrich (HPA004773). Actin antibody was from Sigma-Aldrich (A1978), Histon H3 antibody (D1H12) from Cell Signalling (4499L), Lamin B from Progen (61047C) and GAPDH from GeneTex (GTX627408). Chemiluminescence analysis was done using lumi-light (Roche), dura or femto reagent (Pierce) in the LAS 4000 detection system (GE Healthcare).

## SUPPLEMENTARY FIGURES AND TABLES


