# Supplementary material for: CXorf61 is a target for T cell based immunotherapy of triple-negative breast cancer
Source: Oncotarget. 2015 Jul 29;6(28):25356–67. doi: 10.18632/oncotarget.4516 (PMC4694836; doi:10.18632/oncotarget.4516)
Supplement: Supplementary file 1 [file oncotarget-06-25356-s001.pdf]

SUPPLEMENTARY FIGURES AND TABLES

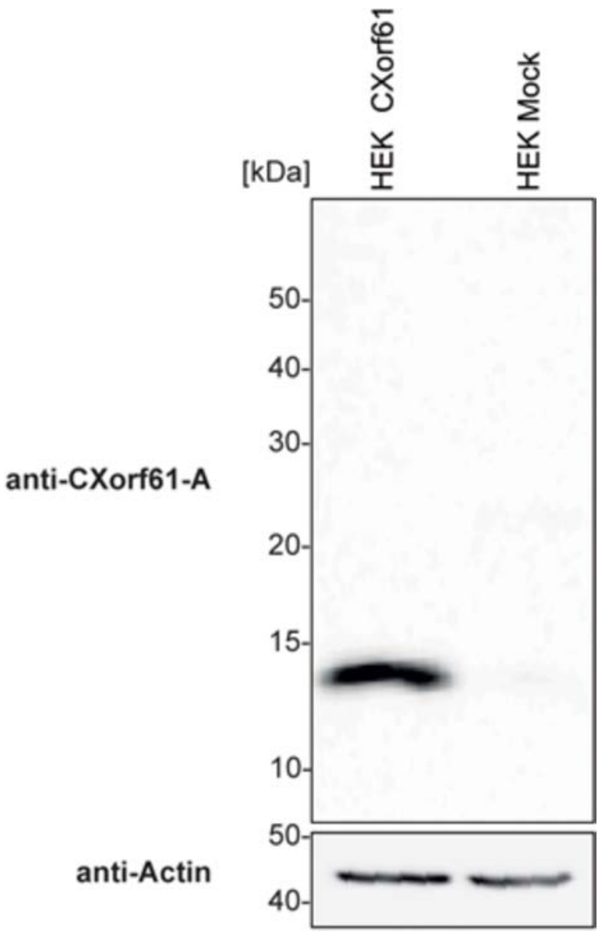

Supplementary Figure S1: Validation of a polyclonal anti-CXorf61 antibody (anti-CXorf61-A). An anti-CXorf61 antibody was raised in rabbit and affinity purified. The antibody was validated using HEK 293T cells transfected with a plasmid containing CXorf61 (HEK CXorf61) or vector alone (HEK Mock). CXorf61 signal was compatible with the predicted size of about 13kDa only in HEK CXorf61 cells. No background was observed in the mock control. Actin was used as loading control.

Supplementary Table S1: Characteristics of TNBC patients and CXorf61 expression

ID	Age at surgery	Histology	Grade	Stage, TNM				CXorf61Relative expression (a)	%positive cells (b)
1	52	ductal	G2	IIA	T2	N0	M0	715	n.a
2	54	ductal	G2	IIB	T3	N0	M0	381112	n.a
3	52	ductal	G3	IIIA	T3	N1	M0	287474	n.a
4	60	ductal	G3	IIA	T2	N0	M0	2190	n.a
5	43	ductal	G3	I	T1	N0	M0	146135	n.a
6	65	ductal	G2	IIIB	T4	N1	M0	112447	n.a
7	62	ductal	G1	IIB	T3	N0	M0	1	n.a
8	54	ductal	G3	IIA	T2	N0	M0	557492	n.a
9	44	ductal	G2	IA	T1	N0	Mx	49290	n.a
10	57	ductal	G3	IIB	T2	N1	M0	116133	n.a
11	76	ductal	G3	IIIA	T3	N2	M0	598847	n.a
12	35	ductal	G3	IIIA	T3	N2	M0	1	n.a
13	83	ductal	G3	IIB	n.a	n.a	n.a	1	n.a
14	51	ductal	G3	IIB	T2	N1	M0	1	n.a
15	49	ductal	G3	IIIA	T2	N2	M0	724174	n.a
16	36	ductal	G2	IIA	T2	N0	M0	1	n.a
17	n.a	ductal	G2	IIA	T2	N0	M0	1	n.a
18	53	ductal	G2	IIA	T2	N0	M0	23168	n.a
19	31	ductal	G3	IIA	T2	N0	M0	757530	0
20	52	ductal	G2	IIA	T2	N0	M0	664228	n.a
21	52	ductal	G3	IIIA	T2	N2	M0	179153	n.a
22	57	ductal	G3	IIA	T2	N0	M0	45779	n.a
23	49	ductal	G3	IIA	T2	N0	M0	61515	n.a
24	40	ductal	G2	IIA	T2	N0	M0	1	n.a
25	64	lobular	G2	IIIA	T2	N2	M0	1	n.a
26	85	ductal	G3	IIA	T2	N0	M0	159432	n.a
27	43	ductal	G2	IIIC	T3	N3	M0	3813	n.a
28	73	lobular	G3	IIIC	T3	N3	M0	1	n.a
29	57	ductal	G3	IIA	T2	N0	M0	750	n.a
30	49	ductal	G3	IIIC	T2	N3	M0	1	n.a
31	74	ductal	G2	IIA	T2	N0	M0	3912	n.a
32	47	ductal	G3	IIA	T2	N0	M0	272563	n.a
33	50	ductal	G3	IIA	T2	N0	M0	525823	n.a
34	67	ductal	G3	IIA	T2	N0	M0	334475	n.a
35	54	ductal	G3	IIA	T2	N0	M0	245913	70

(*Continued*)

ID	Age at surgery	Histology	Grade	Stage, TNM				CXorf61Relative expression (a)	%positive cells (b)
36	56	ductal	G2	IIA	T2	N0	M0	3179	n.a
37	83	ductal	G2	IIA	T2	N0	M0	474223	n.a
38	50	n.a	G2	IIB	T3	N0	M0	3240	n.a
39	82	ductal	G3	IIIB	T4	N0	M0	111863	n.a
40	45	n.a	G3	IIA	T2	N0	M0	322871	60
41	68	ductal	G3	IIIA	T2	N2	M0	235451	n.a
42	42	ductal	G3	IIA	T2	N0	M0	213918	n.a
43	48	ductal	G3	IIA	T2	N0	M0	292191	n.a
44	55	ductal	G3	IIA	T2	N0	M0	660	90
45	62	ductal	G3	IIA	T2	N0	M0	63424	n.a
46	40	ductal	G3	IIA	T2	N0	M0	149381	n.a
47	80	ductal	G3	IIA	T2	N0	M0	194818	30
48	32	n.a	G3	IIA	T2	N1	M0	n.a	0
49	43	n.a	G3	IIB	T2	N1	M0	n.a	0
50	55	n.a	G3	IIA	T2	N0	M0	1	n.a
51	48	n.a	G3	IIA	T2	N0	M0	169733	n.a
52	73	n.a	n.a	IIB	T2	N1	M0	1	0
53	43	n.a	G3	IIA	T2	N0	M0	269923	n.a
54	53	n.a	G3	IIIC	T2	N3	M0	1	80
55	49	n.a	G3	IIIC	T2	N3	M0	417952	n.a
56	41	n.a	n.a	IIB	T2	N1	M0	n.a	70
57	74	ductal	G3	IIA	T2	N0	M0	n.a	0
58	80	ductal	G2	IIA	T2	N0	M0	n.a	40
59	50	ductal	G3	IA	T1	N0	M0	n.a	10
60	37	ductal	G2	IIB	T2	N1	M0	n.a	0
61	65	ductal	G2	IIA	T2	N0	M0	n.a	10
62	69	ductal	G3	IIB	T2	N1	Mx	n.a	60
63	63	ductal	G3	IIA	T2	N0	M0	n.a	70

(a) Relative expression of CXorf61 according to qRT-PCR. (b) % of tumor cells expressing CXorf61 in Immunohistochemistry. (n.a) not available. Stage is according to the International Union Against Cancer UICC.

Supplementary Table S2: 9-mer peptides predicted to bind to HLA-A*0201 by the SYFPEITHY algorithm

Sequence	Position	Score
KLVELEHTL	90–98	27
ILNNFPHSI	66–74	25
YLLLASSIL	4–12	24
RILVNLSMV	79–87	24
LLASSILCA	6–14	23
NLSMVENKL	83–91	23
